# Recomendaciones en la medida de esteroides sexuales. Un paso más en la revolución silenciosa de la espectrometría de masas

**DOI:** 10.1515/almed-2023-0021

**Published:** 2023-03-09

**Authors:** Joan Lopez Hellin

**Affiliations:** Servicio de Bioquímica Clínica, Hospital Universitari Vall d’Hebron. Vall d’Hebron Barcelona Hospital Campus Barcelona, España

La medida de esteroides sexuales mediante espectrometría de masas ha demostrado ser superior a las técnicas de inmunoanálisis convencionales en algunos casos concretos como la medida de concentraciones bajas o ante la presencia de interferentes (recomendaciones publicadas en este número [1]). Este no es un fenómeno aislado, sino que en otras mediciones se observa el mismo fenómeno: cuando las técnicas convencionales de inmunoanálisis llegan a su límite, la espectrometría de masas toma el relevo. Un ejemplo claro es la medida de inmunosupresores, donde la técnica de espectrometría de masas se está imponiendo al inmunoanálisis. ¿A qué se debe esto?

La espectrometría de masas a la que nos referimos para estas mediciones es, hablando con propiedad, la cromatografía líquida con espectrometría de masas en tándem (LC-MS/MS) con dilución isotópica. Sobre el papel es la mejor técnica de análisis cuantitativo posible. Es tan perfecta que, bien manejada, no sólo es un método de referencia, sino que se convierte en lo que se considera por la IUPAC un método definitivo, una medida que no puede mejorarse. No es una medida indirecta basada en una propiedad derivada, como por ejemplo la emisión de luz provocada por la unión o no de un anticuerpo a una sustancia, sino que es una medida directa de una propiedad física básica de la molécula a analizar: su masa. Además de tener una especificidad casi absoluta y una muy buena sensibilidad, que aumenta continuamente con los progresos técnicos de los equipos, la técnica de la dilución isotópica permite una cuantificación inmejorable. Y por si esto no fuera suficiente, LC-MS/MS es un método prácticamente universal, ya que permite cuantificar casi cualquier molécula, de glucosa a proteínas, pasando por fármacos. Para finalizar con sus cualidades, al ser un sistema de análisis universal, permite la medida simultanea de varios analitos relacionados sin un aumento de coste importante, lo que permite medir sobre la misma muestra paneles amplios de hormonas, fármacos, biomarcadores, etc. [2].

La pregunta es obvia: si es una técnica tan maravillosa, ¿por qué no se ha implementado ya en todos los laboratorios? Lamentablemente existen algunos factores que limitan su uso. En primer lugar, los equipos de LC-MS/MS son muy complejos, poco o nada automatizados y necesitan una “reingeniería” cada vez que se cambia de método. Esto hace que requieran personal muy cualificado, tanto técnico para el proceso de muestras, monitoreo del proceso y mantenimiento, como facultativo para poner a punto los equipos y métodos, detectar y corregir problemas y analizar los resultados. En segundo lugar, aunque los reactivos que consumen no son caros, el equipo sí lo es, y se necesita una fuerte inversión inicial en hardware, más un gasto anual importante en el mantenimiento de los equipos. En último lugar, la velocidad de proceso de muestras que, aunque individualmente es alta, globalmente se degrada al tener que procesar las muestras secuencialmente, y no en paralelo como en los equipos de inmunoanálisis, no pudiendo competir en velocidad con un analizador de los laboratorios automatizados [3]. Todos estos inconvenientes provocan un temor lógico a instalar la tecnología de espectrometría de masas en laboratorios clínicos, especialmente cuando no se dispone de personal preparado con experiencia previa en este campo, lo que de momento sigue siendo un requerimiento prácticamente imprescindible para tener éxito en la implementación de análisis por LC-MS/MS.

Sin embargo, los progresos técnicos y la actitud de la industria están cambiando esta situación, incrementando las ventajas y eliminando los inconvenientes de la espectrometría de masas, de manera que cada vez más laboratorios se deciden a dar el paso de instalar este tipo de analizadores [4]. Los equipos LC-MS/MS son cada vez más sensibles, rápidos y sencillos de manejo. Los fabricantes de equipos de espectrometría masas, tradicionalmente dedicados a la industria y a la investigación, están entendiendo mejor las necesidades clínicas y están apostando por equipos más sencillos y automatizados y, sobre todo, por servicios de mantenimiento adaptados a los tiempos y necesidades del laboratorio clínico, adecuando el soporte a usuarios que no han tenido una experiencia previa importante en espectrometría de masas. Todo esto va facilitando el uso de la espectrometría de masas en el laboratorio clínico, pero la dificultad sigue siendo grande. No obstante, es necesario señalar que, aunque el esfuerzo para el cambio es grande, la recompensa es enorme. La calidad y fiabilidad de los resultados mejora muy significativamente y, en nuestra experiencia con inmunosupresores, el cambio a la espectrometría de masas es aceptado con entusiasmo por los médicos, que difícilmente aceptarían volver al inmunoanálisis.

Este es el presente, pero el futuro a corto plazo aparece aún más brillante, ya que las empresas tradicionales del diagnóstico clínico se están abriendo a considerar incluir espectrómetros de masas en sus equipos de análisis. Esta puede ser la auténtica revolución de las masas en el laboratorio clínico: analizadores LC-MS/MS adaptados al laboratorio central automatizado, que funcionen como analizadores convencionales, plenamente automatizados e integrados, sin necesitar profesionales especialmente formados ni complejas operaciones de puesta a punto, análisis o mantenimiento. No para sustituir al inmunoanálisis, sino para complementarlo donde se requiera la sensibilidad o la especificidad que este no puede aportar y sacrificando velocidad de proceso y quizás coste: inmunosupresores, estradiol y testosterona a baja concentración o con interferentes, vitamina D en casos especiales, confirmación de drogas de abuso, medida de ciertos antimicóticos, antibióticos, antiepilépticos que ahora hay que analizar por cromatografía líquida.

La recomendación de usar LC-MS/MS en la medición de esteroides sexuales en determinadas situaciones es un paso más en el camino lento pero irreversible hacia la implantación de la espectrometría de masas en el laboratorio clínico, donde previsiblemente convivirán los equipos de masas automáticos integrados en los laboratorios centrales automatizados con los equipos LC-MS/MS convencionales, reservados para laboratorios de referencia, donde se analizarán moléculas para las que no se ha desarrollado el análisis automático, ya sea por novedad o por falta de interés comercial. Mi sensación es que estamos viviendo un momento similar a cuando los únicos métodos de inmunoanálisis eran RIA, western blot y ELISA, y empezaba a oírse hablar de los analizadores inmunoquímicos. En aquel momento era difícil imaginar lo que ahora es rutina: un laboratorio central con analizadores bioquímicos y inmunoquímicos integrados trabajando a alta velocidad. Muy probablemente en un futuro no muy lejano veremos laboratorios centrales con equipos de bioquímica, inmunoquímica y espectrometría de masas integrados, cada uno con su función específica, y mejorando muy significativamente la capacidad analítica del laboratorio clínico.
